# Psychometric properties and normative values of the revised demoralization scale (DS-II) in a representative sample of the German general population

**DOI:** 10.1186/s12888-023-05187-9

**Published:** 2023-09-20

**Authors:** Markus Ramm, Johanna Jedamzik, Philipp Lenz, Anileeta Poopana, Gereon Heuft, Rupert Conrad

**Affiliations:** 1 Department of Psychosomatic Medicine and Psychotherapy, University Hospital Münster, Albert-Schweitzer-Campus 1, 48149 Münster, Germany; 2 West German Cancer Center, University Hospital Münster, Münster, Germany; 3 Institute of Palliative Care, University Hospital Münster, Münster, Germany; 4 LWL Hospital Münster, Münster, Germany

**Keywords:** Cancer, Demoralization, General population, Palliative, Psycho-oncology

## Abstract

**Background:**

Demoralization is a clinically relevant syndrome in chronic diseases. The demoralization scale (DS-II) was recently developed as an economic screening tool in clinical populations. Main aim of this study was to provide normative data of DS-II scores in the general population.

**Methods:**

We developed a new German version, the DS-II Münster, and tested internal consistency as well as the previously proposed two-factor structure with confirmatory factor analyses. The DS-II was applied in a household survey of the general population. Associations between DS-II scores and age, gender and other sociodemographic variables were explored.

**Results:**

The final sample consisted of *N* = 2471 participants (mean age = 49.8 years, range: 18–96; 50.1% men, 49.8% women). The DS-II Münster showed nearly excellent internal consistency. The model fit indices of the two-factor structure were not superior to those of the one-factor model. Mean scores of the DS-II were as follows. Total score: M = 3.76 (SD = 5.56), Meaning and Purpose subscale: M = 1.65 (SD = 2.77), Distress and Coping Ability subscale: M = 2.11 (SD = 3.02). DS-II scores were increased in women with an effect size of Cohen’s d = 0.19. An age-related increase was specifically found for the Meaning and Purpose subscale (d = 0.21).

**Conclusions:**

The study provides normative values of the DS-II with respect to age and gender in the general population to facilitate interpretation of DS-II scores in clinical samples. A DS-II total score > 5 is suggested as a cut-off value. The findings further our understanding of significant symptom burden that was previously suggested in young patients with cancer.

**Supplementary Information:**

The online version contains supplementary material available at 10.1186/s12888-023-05187-9.

## Background

Demoralization is defined as a perceived inability to cope with a stressor, resulting in feelings of hopelessness and helplessness, a sense of incompetence or failure, and loss of meaning and purpose [1, 2]. It is a mental state commonly observed in palliative care patients who suffer from existential distress [3, 4]. Indeed, demoralization syndrome has frequently been studied in the context of patients with cancer at the end of life [5, 6] in which clinically significant demoralization rates range between 13 and 18% [7]. Demoralization syndrome has attracted increasing research interest also in the context of other chronic physical illnesses [8], including Parkinson disease [9], eating disorders [10], pain [11] and organ transplantation [12, 13].

Although demoralization is closely related to depression previous studies indicate that demoralization and anhedonia are distinct entities which overlap only in the more severe forms of demoralization [14, 15]. Moderate to medium levels of demoralization more strongly reflect a failure to cope and adjust to a stressor, which is the key concept of adjustment disorder in the ICD-11 definition [16]. In advanced patients with cancer, demoralization increases symptom burden and causes a feeling that one is a burden to others [17]. Demoralization represents a serious mental health concern as it is associated with poor quality of life [18] and is a risk factor for suicide independent from its association with depression [18–20].

In recent systematic reviews [7, 21], the most frequently used self-report measure of demoralization was the 24-items Demoralization Scale [DS-I; [2]] which has been translated to several languages including German [22]. It has been applied in several studies with clinical samples, particularly in patients with oncological diseases or other progressive diseases (for reviews see Wozniewicz, Cosci [21], Robinson et al. [7], Robinson et al. [4]). However, shortcomings of this 24-items scale such as the high burden for palliative care patients, an inconsistent factor structure of four to five factors, and the reversed items that may reduce reliability [2, 23] limited clinical applicability, so that a revised version of the DS-I, the Demoralization Scale-II (DS-II) was developed [24, 25]. An exploratory factor analysis yielded a two-factorial structure in palliative patients [24]. Results suggest that the simplified and psychometrically improved DS-II is a more feasible measure of demoralization for research and clinical application.

Beyond the initial validation study, done by Robinson et al. [24], a limited number of studies with the revised DS-II have yet been conducted. So far, a Spanish version of the DS-II was applied in *N* = 150 patients with advanced cancer in Spain and Latin America [26] and a Chinese version of the DS-II was validated in *N* = 296 patients with cancer [27]. Ignatius, De La Garza [28] provided data from a large sample (*N* = 922) of adult patients with cancer seen in an outpatient psychiatric oncology clinic in the USA. In 2021, the DS-II was translated into German and applied in a large sample (*N* = 620) of patients with advanced cancer [29]. This German version showed good internal consistency, but instead of the two-factor structure observed in the original version of the DS-II [24], data challenged the two-factor structure [29]. The authors decided not to report another factor structure to contribute to international comparability of the DS-II and suggested a one-dimensional structure of the German version of the DS-II.

Notably, interpretation of DS-II scores in these studies in terms of symptom burden requires the knowledge of normative values of the general population. For this reason, empirical data from DS-I scores in the German general population have recently been published [30]. However, to the best of our knowledge, for the revised DS-II, normative values have not yet been provided, limiting applicability of the revised DS-II as a screening tool in research and clinical practice.

As the existing German version of the DS-II [29] failed to meet criteria for a good model fit, an alternative German version of the DS-II, the “Demoralization Scale-II Münster”, was developed and tested. Aim of the present study was to (1) test its internal consistency and factorial structure in a large representative sample of the norm population, (2) provide empirical data of demoralization in the general population, and (3) explore the impact of age, gender and other sociodemographic parameters on the DS-II scores.

## Methods

### Subjects

Data were acquired between March and May 2022 by a demography consulting company (USUMA, Berlin, Germany) as part of a broader German household survey. Participants were carefully selected as follows: Proportionally to the distribution of private households, the country was separated into 258 areas representing all regions in Germany. After the selection of a sample point, houses, households and household members were chosen randomly. In total, *n* = 6192 households in 258 areas were selected (*n* = 6188 were valid), approaching a representative sample of the German general population. The survey was conducted as a face-to-face interview with the participants. The interviewer provided information about the study and handed out self-report questionnaires. *N* = 2522 (41,2%) subjects aged ≥ 16 years gave written informed consent to participate. In our analyses, participants < 18 years (N = 44) were excluded. Furthermore, subjects with at least two items missing for one of the two subscales of the DS-II (*N* = 7) were also discharged, resulting in a final sample of *N* = 2471. In case there was not more than one missing item of each subscale, missing values were replaced by the rounded mean of the valid items.

### Measure

#### Demoralization Scale (DS-II) Münster

The DS-II is a recently developed and validated self-report questionnaire with 16 items that are rated on a three-point Likert scale (0 = never; 1 = sometimes; 2 = often), thus total DS-II scores range from 0–32. In comparison to the original DS-I, the DS-II is more user-friendly due to fewer items and only three answer options instead of five. It comprises two subscales with eight items each, the “Meaning and Purpose subscale” and the “Distress and Coping Ability subscale” (Items of the DS-II and its corresponding subscale are presented in Table 2). In the original study of the DS-II with 211 palliative care patients, internal consistency was high (α = 0.89 for the total scale, α = 0.84 for the Meaning and Purpose subscale and α = 0.82 for the Distress and Coping Ability subscale). Convergent validity was proved with measures of psychological distress and quality of life. With respect to discriminant validity, divergence was found between DS-II scores and depression assessed by the PHQ-9 at low levels of the construct [25]. Robinson et al. [25] proposed the following clinical cut-off criteria: 0–3 (“low demoralization”), 4–10 (“moderate demoralization”) and ≥ 11 (“high demoralization”). For the current study, we translated the original DS-II to German according to state-of-the-art translation procedures [31]. Forward translation was performed by two bilingual and bicultural translators with knowledge of the research field. The translators discussed and agreed upon a final German version. Back-translation was performed by two translators (who were English native speakers). This English version was compared to the original DS-II and discrepancies were discussed until agreement was reached. Before beginning the survey, we tested this version on a small sample of 10 subjects which did not report problems in understanding. The final DS-II version was named “Demoralization Scale-II Münster” and is freely available upon request.

### Statistical analysis

Statistical analysis was carried out with IBM SPSS® Statistics Software (version 28.0, IBM, Armonk, NY). R (version 4.2.1, The R foundation for Statistical Computing, 2022, Vienna, Austria) was used for computing the confirmatory factor analysis (CFA).

We performed CFA using maximum likelihood estimation, testing the previously supposed two-factor structure and a one-factor model, aggregating all 16 items. The models were specified as follows: In the two-factor model, both factors, “Meaning and Purpose” and “Distress and Coping Ability” were correlated. Items loaded on a single latent factor (see Fig. 2). Variances of the latent factors were set to one, resulting in over-identified models with 103 degrees of freedom and 33 free parameters (two-factor model) and 104 degrees of freedom and 32 free parameters (one-factor model). Global model fit was evaluated using several statistics: Chi-square as a measure of exact model fit, approximate fit indices including Root Mean Square Error of Approximation (RMSEA), standardized Root Mean Square Residual (SRMSR), Comparative Fit Index (CFI), Tucker-Lewis Index (TLI) and Bayesian information criterion (BIC). Model fit was evaluated according to the guidelines [32]. Internal consistency of the DS-II total score and the two subscales were measured by Cronbach’s alpha (α).

Separate two-way ANOVAs with “age group” and “gender” as between-subjects factors for the DS-II total scale and both subscales were performed. Gender refers to “gender identity”, distinguishing female, male and non-binary.

Further ANOVAs with age group and gender as covariates were conducted to test for effects of other sociodemographic variables, including partnership (living with a partner vs. living not with a partner), education (without graduation, basic school qualification, middle maturity or similar qualification, technical school, A-level, university education), occupational status (employed, unemployed/short-time work, schooling/ professional training, retired, stay-at-home individuals) and income groups (< 1250€, 1250 – < 2500€, > 2500€). For statistical analyses, subjects with other gender than male/female and those with “other educational qualification” were excluded due to the low sample sizes (*n* < 10).

As there is reasonable skew in the demoralization scores in the general population, we additionally present median and further percentile for the DS-II data. Due to the central limit theorem, in our large sample-size study, an approximation of a normal sampling distribution in the analyzed populations can be presumed. Furthermore, within a fairly balanced design, estimated population variances were comparable (i.e., ratio of the highest to the smallest variance < 1.5) between the analyzed groups for factors “age”, “gender” and “partnership”. *P*-values of all post hoc pairwise comparisons were corrected using Bonferroni procedure. Of note, for the other sociodemographic factors, we had an unbalanced design with different population variances, suggesting that parameter estimation and significance tests of the ANOVAs might be less reliable. To overcome bias, in post hoc tests, Bonferroni corrected p-values and additionally bootstrapped (*n* = 1000 samples; bias corrected and accelerated) 95% confidence intervals (CI) for differences of group means are reported. For all analyses, critical p was set at 0.05. Cohen’s d is given as an effect size parameter for significant effects.

## Results

### Sample characteristics

Table 1 shows sociodemographic characteristics of the analyzed study sample. The group consisted of 50,0% males, 49,8% females and 0.2% non-binary. Mean age was 49.8 years (SD = 17.3; range: 18–96).

**Table 1:** Sample characteristics. M, mean; SD, standard deviation. *N* = 34 subjects did not report if they live with or without a partner. *N* = 14 subjects with other education are not displayed. *N* = 11 subjects did not report occupational status. *N* = 31 subjects did not report income

	**Total (*n* = 2471)**		**Men (*n* = 1237)**		**Women (*n* = 1230)**	
**Age, M(SD)**	49.81 (17.3)		49.66 (17.3)		50.02 (17.3)	
**Age groups**	**N**	**%**	**N**	**%**	**N**	**%**
< 30 years	370	15.0	180	14.6	188	15.3
30 – 39 years	404	16.3	22	17.9	181	14.7
40 – 49 years	432	17.5	205	16.6	226	18.4
50 – 59 years	500	20.2	246	19.9	254	20.7
60 – 69 years	395	16.0	201	16.2	194	15.8
≥ 70 years	370	15.0	183	14.8	187	15.2
**Partnership**						
Living with a partner	1551	62.8	830	67.1	719	58.5
Living without a partner	886	35.9	398	32.2	486	39.5
**Education**						
Without graduation	60	2.4	31	2.5	29	2.4
Basic school qualification	615	24.7	315	25.6	300	24.6
Middle maturity	838	34.0	371	30.1	464	38.1
Ten-class general educational polytechnic secondary school	213	8.6	109	8.9	104	8.5
Technical school	108	4.4	64	5.2	44	3.6
A-level	329	13.3	171	13.9	157	12.9
University education	291	11.8	170	13.8	121	9.9
**Occupational status**						
Employed	1584	64.1	827	66.9	754	61.3
Unemployed/short-time work	100	4.0	55	4.4	44	3.6
Schooling/professional training	115	4.7	47	3.8	68	5.5
Retired	605	24.5	294	23.8	311	25.3
Stay-at-home	56	2.3	8	0.6	48	3.9
**Household income**						
< 1250€	226	9.1	102	8.2	124	10.1
1250- < 2500€	952	38.5	437	35.3	513	41.7
> 2500€	1262	51.1	685	55.4	575	46.7

### Item statistics and internal consistency of the DS-II

Single-item statistics are presented in Table 2. The items with the highest affirmation were item 1 (“There is little value in what I can offer others”; M = 0.49; SD = 0.63) and item 8 (“I feel irritable”; M = 0.40; SD = 0.56) whereas item 14 (“I would rather not be alive”; M = 0.07; SD = 0.30) and item 13 (“I am not a worthwhile person”; M = 0.15; SD = 0.43) received the lowest affirmation. Thus, the range of mean affirmation to the items was 0.07 to 0.49, representing only 14% of the DS-II answer options.

**Table 2:** Item characteristics of the German DS-II Münster. M, mean; SD, standard deviation, subscale 1, Meaning and Purpose subscale; subscale 2, Distress and Coping Ability Subscale; ^a^part-whole-corrected correlation between item and total score; ^b^part-whole-corrected correlation between item and the corresponding subscale

Item number	Item	Subscale	M (SD)	r^a^	r^b^
1	There is little value in what I can offer others	1	0.49 (0.63)	.49	.47
2	My life seems to be pointless	1	0.17 (0.43)	.77	.77
3	My role in life has been lost	1	0.21 (0.48)	.71	.71
4	I no longer feel emotionally in control	2	0.18 (0.42)	.65	.61
5	No one can help me	1	0.18 (0.44)	.74	.73
6	I feel that I cannot help myself	1	0.21 (0.46)	.78	.75
7	I feel hopeless	1	0.17 (0.43)	.76	.75
8	I feel irritable	2	0.40 (0.56)	.55	.57
9	I do not cope well with life	2	0.19 (0.46)	.76	.71
10	I have a lot to regret about my life	2	0.32 (0.56)	.58	.58
11	I tend to feel hurt easily	2	0.32 (0.54)	.64	.67
12	I feel distressed about what is happening to me	2	0.26 (0.52)	.72	.69
13	I am not a worthwhile person	1	0.15 (0.43)	.76	.76
14	I would rather not be alive	1	0.07 (0.30)	.63	.64
15	I feel quite isolated or alone	2	0.23 (0.51)	.70	.65
16	I feel trapped by what is happening to me	2	0.21 (0.49)	.79	.75

Cronbach’s alpha coefficients were α = 0.94 for the total scale, α = 0.89 for the Meaning and Purpose subscale and α = 0.88 for the Distress and Coping Ability subscale. Both subscales were highly correlated (*r* = 0.85).

The part-whole-corrected correlations between each single item and the mean total score were between *r* = 0.49 and *r* = 0.79. For the Meaning and Purpose subscale, part-whole corrected item-scale correlations were mostly above *r* = 0.64, except item 1 (*r* = 0.47). Similar contributions of the items to the Distress and Coping Ability subscale were observed.

### Factor structure of the DS-II

**Fig. 1:**
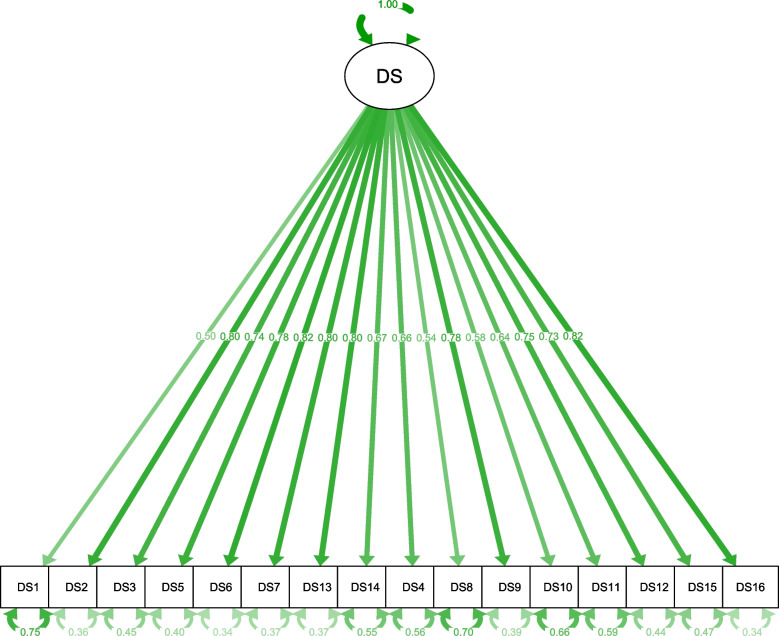
Standardized parameter estimates of the one-factor model. DS, Demoralization Scale-II – total score. Factor structure, factor loadings and residual variances are shown

**Fig. 2:**
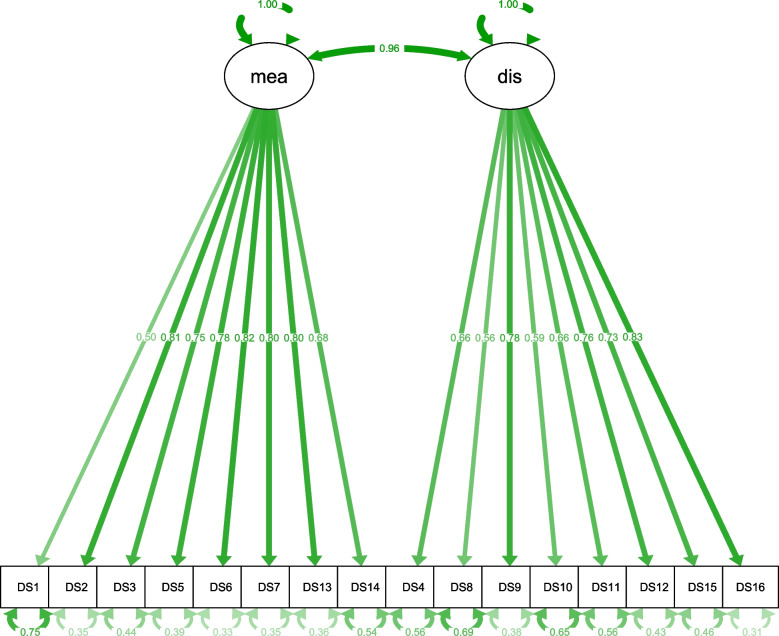
Standardized parameter estimates of the two-factor structure. Mea, factor “Meaning and Purpose” (subscale 1); dis, factor "Distress and Coping Ability” (subscale 2). Factor structure, factor loadings, correlation between latent factors and residual variances are shown

Standardized results of both, the one-factor and the two-factor model estimation, are shown in Figs. 1 and 2.

Standard error, z-value, *p*-value and 95% CI of the standardized parameters are displayed in the supplement (Table S1). Moreover, Table 3 summarizes global model fit indices. Both models revealed similar model fit indices. While SRMR indicated good model fit, RMSEA just missed the cut-off of 0.06. Moreover, CFI/TLE, which should be above 0.95, were close to or above good model fit cut-off value.

**Table 3:** Global fit indices for both models. Χ^2^, Chi square test; df, degrees of freedom; CFI, Comparative fit Index; TLI, Tucker-Lewis Index; RMSEA, Root Mean Square Error of Approximation; SRMR, Standardized Root Mean Squared Residual; CI, confidence interval; SABIC, Sample-size adjusted Bayesian information criterion

	**Χ**^**2**^** (df)**	**CFI**	**TLI**	**RMSEA (90%CI)**	**SRMR**	**SABIC**
1-factor model	1328.1 (104)	0.949	0.941	0.069 (0.066; 0.072)	0.032	30,263
2-factor model	1184.3 (103)	0.955	0.947	0.065 (0.062;0.069)	0.030	30,124

### Normative values

Mean DS-II scores were M = 3.76 (SD = 5.56) for the total scale, M = 1.65 (SD = 2.77) for the Meaning and Purpose subscale and M = 2.11 (SD = 3.02) for the Distress and Coping Ability subscale. Means and standard deviations of the total scale and both subscales separated by gender and age group are shown in the supplement (Table S2). Median and interquartile ranges (IQR) were Md = 1 (IQR = 0–5) for the DS-II total scale, Md = 1 (IQR = 0–2) for the Meaning and Purpose subscale and Md = 1 (IQR = 0–3) for the Distress and Coping Ability subscale.

13.6% of the general population reach DS-II total scores (12.9% for Meaning and Purpose subscale; 13.5% for Distress and Coping Ability subscale) with at least one standard deviation above the mean DS-II value (cut-off = 9.32). With reference to an extreme group design (low demoralization =  < 25^th^ percentile, moderate demoralization = 25^th^ – 75^th^ percentile, high demoralization =  > 75^th^ percentile) as suggested by Robinson et al. [25], 33.7% of the general population correspond to low demoralization (score = 0), 44.3% show moderate (score = 1–5) and 22% have high (score > 5) DS-II scores.

Using the proposed clinical cut-off values in the original DS-II study [25], 69.7% of the general population reach values of 0–3, 18.4% show scores between 4–10 and 11.9%score ≥ 11 on the DS-II total scale.

For ease of clinical utility, the median, 75^th^, 90^th^ and 95^th^ percentile for the DS-II total score, separated by gender and age group, are reported in Table 4. The data represent the percentage of the population that reaches the indicated score or below on the DS-II.

**Table 4:** Normative raw values of the DS-II total score depending on gender ^(a)^ and age group. DS-II, Demoralization Scale-II; y, years. (a) Other gender than men or women are not shown due to low number of subjects

	**Total sample**	**Men**						**Women**					
**DS-II percentile**	**18-96y**	**18-29y**	**30-39y**	**40-49y**	**50-59y**	**60-69y**	** ≥ 70y**	**18-29y**	**30-39y**	**40-49y**	**50-59y**	**60-69y**	** ≥ 70y**
50% (Median)	1	1	1	1	2	1	2	2	2	1	2	2	3
75%	5	3	3	3	4	4	5	5	6	5	6	7	8
90%	12	9	8	7	13	15	11	12	13	12	14	13	15
95%	16	15	15	13	15	18	14	16	17	17	17	16	18

### Impact of gender and age group on DS-II scores

**Fig. 3:**
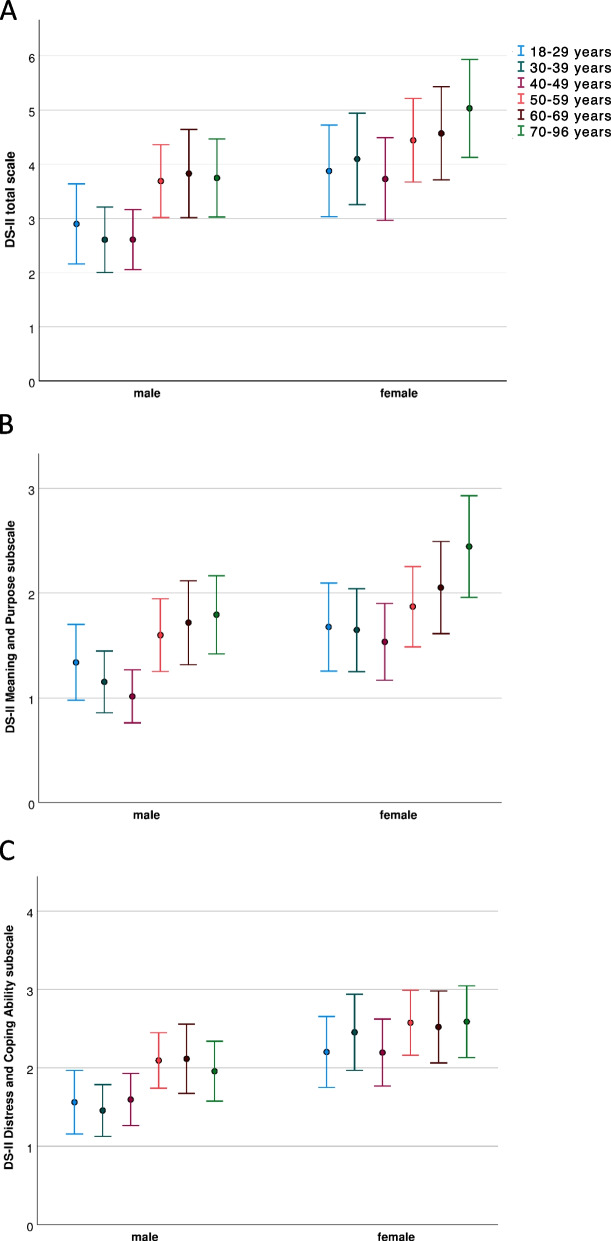
Mean and 95% confidence intervals of DS-II scores in different age groups, separated for men and women. (a) DS-II total scale, (b) Meaning and Purpose subscale, (c) Distress and Coping Ability subscale. DS-II, Demoralization scale-II

Means and 95% CI of DS-II total and subscale scores, respectively, related to age group and gender are displayed in Fig. 3.

Two-way ANOVAs revealed significant main effects of age group (total scale: F_(5,2455)_ = 3.52; *p* = 0.004; d = 0.17; Meaning and Purpose subscale: F_(5,2455)_ = 5.26; *p* < 0.001; d = 0.21; Distress and Coping Ability subscale: F_(5,2455)_ = 2.24; *p* = 0.048; d = 0.14) and gender (total scale: F_(1,2455)_ = 22.42; *p* < 0.001; d = 0.19; Meaning and Purpose subscale: F_(1,2455)_ = 15.2; *p* < 0.001; d = 0.16; Distress and Coping Ability subscale: F_(1,2455)_ = 26.55; *p* < 0.001; d = 0.21). However, there was no age group x gender interaction, neither for the total scale (F_(5,2455)_ = 0.3; *p* = 0.9) nor for the Meaning and Purpose subscale (F_(5,2455)_ = 0.28; *p* = 0.92) and the Distress and Coping Ability subscale (F_(5,2455)_ = 0.47; *p* = 0.80).

Demoralization scores were significantly higher in females compared to males, for the total scale as well as for both subscales. Higher DS-II total scores in females (M_diff_ = 1.06; 95% CI 0.63, 1.48; *p* < 0.001) compared to males were consistent over all age groups. Notably, DS-II scores in younger females (< 50 years) roughly level the scores from older males (> 50 years).

With respect to the main effect of age, for the total scale, pairwise comparisons of age groups showed that subjects older than 70 years reported increased DS-II scores compared to subjects of age groups 40–49 years (M_diff_ = 1.20; 95% CI 0.43, 1.93; *p* = 0.033) and 30–39 years on a trend level (M_diff_ = 1.12; 95% CI 0.31, 1.87 *p* = 0.075). For the Meaning and Purpose subscale, we found that the age group ≥ 70 years scored M_diff_ = 0.61 (95% CI 0.19, 01.01; *p* = 0.039) points higher than the age group < 30 years, M_diff_ = 0.75 (95% CI 0.38, 1.14; *p* = 0.003) points higher than subjects 30–39 years and M_diff_ = 0.83 (95% CI 0.43, 01.21; *p* < 0.001) points higher than age group 40–49 years. Subjects between 60–69 years showed increased scores (M_diff_ = 0.59; 95% CI 0.21, 0.97; *p* = 0.030) compared to the age group 40–49 years. For the Distress and Coping Ability subscale, pairwise comparisons of age groups did not yield significant group differences (all *p* > 0.3).

Summarizing the effect of age group on DS-II scores, the results show that the oldest age group (≥ 70 years) reported highest demoralization while the lowest scores were found in the age group 40–49 years. Specifically in the Meaning and Purpose subscale there is an age-related gain, with low scores in younger subjects (18–49 years) that begin to increase at the age of 50 years, reaching a peak value at age group ≥ 70 years.

### Effects of other sociodemographic variables

A partnership protected against demoralization as assessed by the DS-II total scale (F_(1,2429)_ = 19.39; *p* < 0.001; d = 0.18; living with a partner: M = 3.32; SD = 5.11; living without a partner: M = 4.44; SD = 6.13), the Meaning and Purpose subscale (F_(1,2429)_ = 19.53; *p* = 0.002; d = 0.18; living with a partner: M = 1.43; SD = 2.51; living without a partner: M = 1.98; SD = 3.01) and the Distress and Coping Ability subscale (F_(1,2429)_ = 16.45; *p* < 0.001; d = 0.17; living with a partner: M = 1.89; SD = 2.80; living without a partner: M = 2.46; SD = 3.29). Subjects living with a partner reported M_diff_ = -1.01 (95% CI -1.49, -0.55; *p* < 0.001) DS-II points less than those living not with the partner.

Education had a significant protective effect on DS-II total scale (F_(6,2441)_ = 3.01; *p* = 0.006; d = 0.17) as well as on the Meaning and Purpose subscale (F_(6,2441)_ = 3.07; *p* = 0.005; d = 0.17) and the Distress and Coping Ability subscale (F_(6,2441)_ = 2.84; *p* = 0.009; d = 0.17). Notably, subjects without graduation reported a mean increase of M_diff_ = 2.3 (95% CI 0.68, 4.04; *p* = 0.044) DS-II points compared to people with basic school qualification and scored even M_diff_ = 2.9 (95% CI 1.31, 4.66; *p* = 0.004) points higher than those with a university degree. Interestingly, increased academic qualification did not further reduce demoralization scores.

Higher household income predicted less demoralization on the DS-II total scale (F_(2,2431)_ = 68.06; *p* < 0.001; *d* = 0.47) as well as on the Meaning and Purpose subscale (F_(2,2431)_ = 68.07; *p* < 0.001; *d* = 0.47) and the Distress and Coping Ability subscale (F_(2,2431)_ = 57.98; *p* < 0.001; *d* = 0.44). The lowest income group showed increased DS-II scores compared to the middle-income group (M_diff_ = 3.8; 95% CI 2.74, 4.80; *p* < 0001) and compared to the highest income group (M_diff_ = 4.6; 95% CI 3.6, 5.7; *p* < 0.001). The high-income group reported less demoralization than the middle-income group (M_diff_ = -0.8; 95% CI -1.21, -0.33; *p* = 0.002).

Occupational status had a significant impact on the DS-II total scale (F_(4,2449)_ = 27.18; *p* < 0.001; d = 0.42) as well as on the Meaning and Purpose subscale (F_(4,2449)_ = 28.8; *p* < 0.001; d = 0.43) and the Distress and Coping Ability subscale (F_(3,2430)_ = 22.13; *p* < 0.001; d = 0.38). Unemployed people or those with short-time work showed increased DS-II total scores compared to any other occupational status group, i.e., they reported M_diff_ = 5.6 (95% CI 4.02, 7.14; *p* < 0.001) DS-II points more than employed subjects, M_diff_ = 4.9 (95% CI 2.85, 6.80; *p* < 0.001) DS-II points more than subjects during schooling/professional training, M_diff_ = 4.6 (95% CI 2.79, 6.31; *p* < 0.001) DS-II points more than retired people and M_diff_ = 3.7 (95% CI 1.10, 6.21; *p* < 0.001) points more than stay-at-home individuals.

## Discussion

In the present study, we translated the recently developed DS-II to German language and applied this version (DS-II Münster) in a representative sample of the German general population. This is the first study that provides normative values of the DS-II with respect to age and gender based on *N* = 2471 participants which facilitates appropriate interpretation of DS-II scores in clinical populations. Main results were as follows: The DS-II showed high internal consistency. CFA results yielded nearly good model fit for both, the one-factor and the two-factor solution. Mean DS-II scores in the general population were low, predictors of DS-II scores were gender, age group, income, occupational status, partnership and education.

First, we aimed to test internal consistency and the factor structure of this first-time applied German DS-II Münster. We found that all items substantially contributed to the total scale and the subscales, with item-test correlations that were all above *r* = 0.47. Cronbach’s alpha was excellent for the total scale (α = 0.94) and good for the proposed subscales. These coefficients are similar to those obtained in the previous German DS-II version applied in patients with cancer [29] and higher than reliability scores of the DS-II version applied the original DS-II study [24] and in patients from Spain and Latin America [26]. Notably, Cronbach’s alpha coefficient is exactly the same in the DS-I total scale [30] and in the DS-II total scale. Thus, internal consistency of the DS-II Münster is satisfactory.

Based on data from patients with advanced cancer, the original DS-II study suggested a two-factor structure with 36% shared variance between the subscales [24]. Instead, a previous study using another German version of the DS-II challenged the two-factor structure and suggested the one-dimensional solution [29]. In the present study, for both models, the CFA revealed model fit indices near to the cut-off values of good model fit. Against our expectations, data based on the alternative German DS-II, the DS-II Münster, indeed supported the notion that the two-factor structure is not clearly preferred over the one-factor model, in line with the previous German study in patients with cancer [29]. Thus, the factor structure of the DS-II seems less consistent between different languages and/or populations. As there is about 72% shared variance between both subscales, interpreting the DS-II as a unidimensional scale might be a practicable solution. One possible explanation might be that the two-factor structure of the DS-II is suitable for patients in a palliative setting, whereas “Meaning and Purpose” on the one hand and “Distress and Coping Ability” on the other hand greater correlate in other populations. This highlights the need for further research on factors that have an impact on the DS-II factor structure. Importantly, the CFA results also suggest that there is a factor-structure that might even better fit the data set, which however would impact the comparability between DS-II studies. Accordingly, it seems reasonable to report results not only for the one-factor model but also for the previously proposed two-factor-solution.

One of the main results was that mean demoralization scores as assessed by the DS-II are particularly low in the general population, i.e. about two to three times lower than average DS-II scores in clinical populations, including patients with cancer and other progressive diseases in palliative care [24], adult outpatient patients with cancer in the USA [28], patients with cancer in a Chinese cancer hospital [27] and a heterogenous sample of advanced patients with cancer from different German cancer centers [29]. Based on our results, we suggest a DS-II total score > 5 as a first reasonable cut-off value which would detect about 50–75% of patients with cancer as demoralized, i.e., roughly corresponding to those that were previously classified as “moderately demoralized” (Wu et al. [27]: score > 7; Ignatius, De La Garza [28]: score > 4; Robinson et al. [25]: score > 3). Besides the lower cut-off value (> 5), a total score ≥ 12 might be useful as a second cut-off value as this refers to the highest 10% of the scores in the general population. Interestingly, DS-II scores are well in line with the previous study using the first version of the DS (DS-I) in a representative sample of the German general population. In this study, 13.5% of participants scored one standard deviation above the mean [30] whereas in our sample 13.6% of the subjects reached values one standard deviation above the mean (score ≥ 9.32). In sum, our data support previous conclusions from studies with patients with cancer that the shortened DS-II sufficiently differentiates between clinically relevant demoralization and normative demoralization in the general population.

Another aim of the study was to investigate effects of sociodemographic variables on DS-II scores. Women reported increased DS-II scores compared to men, however the effect was small (d = 0.19) which is in accordance with previous results in the general population using the DS-I [d = 0.12; Quintero Garzón et al. [30]]. This finding was expected as female gender increases the risk for mental health problems (for a review see Kiely et al. [33]). Notably, gender did not interact with age groups in our study, whereas Quintero Garzón et al. [30] described that in the oldest age-group (≥ 70 years) the gender-difference greatly increased, with old women being more demoralized than old men. This result was not replicated in the present study, cautiously suggesting that female gender might be a moderate and lifelong risk factor for demoralization that is probably not significantly related to specific age-dependent developmental tasks.

In clinical populations, some studies did not find gender effects [18, 25, 29] whereas others observed that women were more demoralized than men, showing small effect sizes up to d = 0.35 [13, 22, 28]. A plausible interpretation is that gender effects shown in the general population might interact with clinical aspects in patient samples, leading to either aggravated or diminished demoralization scores. This idea is supported by previous findings from Vehling et al. [34] who reported that female gender was associated with higher demoralization only in younger patients receiving palliative treatment whereas the gender effect was absent in patients in curative treatment.

Age significantly affected DS-II total sores, representing a small effect of d = 0.17, with the age group ≥ 70 years reporting the highest and the age group 40–49 years indicating the lowest scores. Notably, an age-related increase was most clearly found for the Meaning and Purpose subscale (d = 0.21) but it was not significant in the Distress and Coping Ability subscale. Values of the Meaning and Purpose subscale increased in the middle-age (> 50 years) until reaching the highest scores in subjects ≥ *70 years.* These findings are in line with results of a meta-analysis based on 70 studies [35] which found a weak age-associated decline of purpose in life, which was small in subjects < 60 years (*r* = -0.065) but became stronger in the older age groups 60–69 years (*r* = -0.14) and > 70 years (*r* = -0.13). The decrease of meaning in life beginning in the middle-age might be explained by a reduced parental involvement or difficulties in developing new goals after previous goals have been achieved [35]. Indeed, striving for goals seems to be important for maintaining meaning in life [36]. More generally, subjects of older age are more likely to face a limited physical condition due to chronic illnesses, changes in relationships and social roles, including widowhood and retirement, and a lower socioeconomic status. These factors are strong predictors of purpose in life [35].

Furthermore, the age-related increase of DS-II scores has substantial implications for clinicians as our findings contradict most previous reports in cancer populations that described a *decrease* of demoralization with increasing age [22, 25, 28, 29]. Thus, we conclude that the age-related decrease of demoralization is indeed specific to patients with cancer. More specifically, younger patients with cancer (< 50 years) are most severely affected by demoralization [e.g., M = 7.77; SD = 6.26; Koranyi et al. [29]] in the light of the DS-II scores of the corresponding age groups of the general population (see Table S2). In contrast, relatively high demoralization in old subjects, i.e., ≥ 70 years, of the general population (M = 4.40; SD = 5.67) is not further increased in patients with cancer of this age group (e.g., M = 4.44; SD = 5.65; Koranyi et al. [29]). In sum, our findings highlight that younger patients with cancer < 50 years should be considered as the age group with the highest symptom burden which is supported by previous results that young patients with cancer are of increased risk for mood disturbances [37, 38].

Of all sociodemographic factors, occupational status (d = 0.42) and income (d = 0.47) showed the largest impact on DS-II scores, including the subscales. First, unemployed subjects or those with short-time work were most demoralized, their scores largely differed from any other occupational status group. This is consistent with several previous reports of a relation between demoralization and occupational status [30, 39–41]. Moreover, it reflects findings that work is an important source of meaning in life through different ways, including the ability to accomplish valued goals, promote supportive social relationships and contribute to broad organizational aims [42]. Second, household income was positively related to DS-II scores as found in previous studies [30, 43, 44]. This relationship might be due to the potential of money to buffer existential anxiety but also the feeling of being valuable might play a role [45]. Third, living with a partner was a protective factor in the present study, consistent with previous findings [22, 28, 29]. Again, a partnership was shown an important source of meaning in life [35]. Lastly, education turned out to be another buffering factor against demoralization as indicated in previous studies [27, 30, 44].

This study has several limitations. Only 41.2% of the selected and valid households participated in the study, which might reduce generalizability, as DS-II scores in non-responders remain unknown. Nevertheless, the sample was representative of the German general population. The results of this study are based on a German survey so that findings are not necessarily valid in other countries. There might be problems with the generalization of the proposed factor structure of the DS-II to different languages and/or populations. Thus, reevaluation of the factor-structure in future studies is encouraged. The German translation used in the present study (DS-II Münster) has not yet been implemented in patient samples so that its validity and the significance of the proposed cut-off values remain to be tested in future studies.

## Conclusions

We conclude that the DS-II Münster applied in the present study is a feasible measure with high internal consistency that is suitable for the use in research and clinical practice. The normative values related to age and gender can be used for comparisons enabling more adequate interpretation in patients. The factor structure of the DS-II seems to be less consistent between versions of the scale and/or different populations. We hope that with this competing German translation further DS-II research is stimulated.

### Supplementary Information

**Additional file 1: Table S1.** Standardized parameter estimates and associated data of both models. * Items DS1, DS2, DS3, DS5, DS6, DS7, DS13, DS14 load on the factor “mea” (Meaning and Purpose). Items DS4, DS8, DS9, DS10, DS11, DS12, DS15, DS16 load on the factor “dis” (Distress and Coping Ability). CI; confidence interval.

**Additional file 2: Table S2.** Demoralization Scale-II mean and standard deviation by age group and gender. M, mean; SD, standard deviation. DS-II, Demoralization Scale-II.

## Data Availability

The datasets used and analyzed during the current study are available from the corresponding author on reasonable request.

## Abbreviations

DS: Demoralization Scale
IQR: Interquartile range
