# Supplementary material for: Psychometric properties and normative values of the revised demoralization scale (DS-II) in a representative sample of the German general population
Source: BMC Psychiatry. 2023 Sep 20;23:685. doi: 10.1186/s12888-023-05187-9 (PMC10512641; doi:10.1186/s12888-023-05187-9)
Supplement: Supplementary file 1 — Additional file 1: Table S1. Standardized parameter estimates and associated data of both models. * Items DS1, DS2, DS3, DS5, DS6, DS7, DS13, DS14 load on the factor “mea” (Meaning and Purpose). Items DS4, DS8, DS9, DS10, DS11, DS12, DS15, DS16 load on the factor “dis” (Distress and Coping Ability). CI; confidence interval. [file 12888_2023_5187_MOESM1_ESM.docx]

| **Item** | **Coefficient** | **Standard error** | **z-value** | **p-value** | **95% CI** |
| --- | --- | --- | --- | --- | --- |
| **One-factor model** |  |  |  |  |  |
| DS1 | 0.50 | 0.02 | 31.78 | <0.001 | 0.47; 0.53 |
| DS2 | 0.80 | 0.01 | 101.89 | <0.001 | 0.78; 0.82 |
| DS3 | 0.74 | 0.01 | 77.33 | <0.001 | 0.72; 0.76 |
| DS4 | 0.66 | 0.01 | 55.50 | <0.001 | 0.64; 0.68 |
| DS5 | 0.78 | 0.01 | 90.41 | <0.001 | 0.76; 0.79 |
| DS6 | 0.82 | 0.01 | 111.27 | <0.001 | 0.80; 0.83 |
| DS7 | 0.80 | 0.01 | 100.04 | <0.001 | 0.78; 0.81 |
| DS8 | 0.54 | 0.02 | 37.11 | <0.001 | 0.52; 0.57 |
| DS9 | 0.78 | 0.01 | 91.41 | <0.001 | 0.76; 0.80 |
| DS10 | 0.58 | 0.01 | 42.06 | <0.001 | 0.56; 0.61 |
| DS11 | 0.64 | 0.01 | 52.06 | <0.001 | 0.62; 0.67 |
| DS12 | 0.75 | 0.01 | 79.29 | <0.001 | 0.73; 0.77 |
| DS13 | 0.80 | 0.01 | 100.48 | <0.001 | 0.78; 0.81 |
| DS14 | 0.67 | 0.01 | 57.14 | <0.001 | 0.65; 0.69 |
| DS15 | 0.73 | 0.01 | 72.53 | <0.001 | 0.71; 0.75 |
| DS16 | 0.82 | 0.01 | 111.11 | <0.001 | 0.80; 0.83 |
| **Two-factor model*** |  |  |  |  |  |
| DS1 | 0.50 | 0.02 | 31.43 | <0.001 | 0.47; 0.53 |
| DS2 | 0.81 | 0.01 | 103.73 | <0.001 | 0.79; 0.82 |
| DS3 | 0.75 | 0.01 | 78.07 | <0.001 | 0.73; 0.77 |
| DS4 | 0.66 | 0.01 | 55.00 | <0.001 | 0.64; 0.69 |
| DS5 | 0.78 | 0.01 | 92.45 | <0.001 | 0.77; 0.80 |
| DS6 | 0.82 | 0.01 | 112.50 | <0.001 | 0.81; 0.84 |
| DS7 | 0.80 | 0.01 | 102.77 | <0.001 | 0.79; 0.82 |
| DS8 | 0.56 | 0.02 | 38.61 | <0.001 | 0.53; 0.59 |
| DS9 | 0.78 | 0.01 | 91.88 | <0.001 | 0.77; 0.80 |
| DS10 | 0.59 | 0.01 | 43.22 | <0.001 | 0.57; 0.62 |
| DS11 | 0.66 | 0.01 | 54.88 | <0.001 | 0.64; 0.69 |
| DS12 | 0.76 | 0.01 | 80.75 | <0.001 | 0.74; 0.78 |
| DS13 | 0.80 | 0.01 | 101.86 | <0.001 | 0.79; 0.82 |
| DS14 | 0.68 | 0.01 | 58.38 | <0.001 | 0.65; 0.70 |
| DS15 | 0.73 | 0.01 | 73.10 | <0.001 | 0.72; 0.75 |
| DS16 | 0.83 | 0.01 | 115.80 | <0.001 | 0.82; 0.84 |

Table S1. Standardized parameter estimates and associated data of both models. * Items DS1, DS2, DS3, DS5, DS6, DS7, DS13, DS14 load on the factor “mea” (Meaning and Purpose). Items DS4, DS8, DS9, DS10, DS11, DS12, DS15, DS16 load on the factor “dis” (Distress and Coping Ability). CI; confidence interval.
