# Supplementary material for: Psychometric properties and normative values of the revised demoralization scale (DS-II) in a representative sample of the German general population
Source: BMC Psychiatry. 2023 Sep 20;23:685. doi: 10.1186/s12888-023-05187-9 (PMC10512641; doi:10.1186/s12888-023-05187-9)
Supplement: Supplementary file 2 — Additional file 2: Table S2. Demoralization Scale-II mean and standard deviation by age group and gender. M, mean; SD, standard deviation. DS-II, Demoralization Scale-II. [file 12888_2023_5187_MOESM2_ESM.docx]

|  | | **DS-II**  **Total score**  Mean (SD) | **DS-II**  **Meaning and purpose**  Mean (SD) | **DS-II**  **Distress and Coping Ability**  Mean (SD) |
| --- | --- | --- | --- | --- |
| **Total sample** | **N = 2471** | **3.76 (5.56)** | **1.65 (2.77)** | **2.11 (3.02)** |
| <30 years | N = 370 | 3.41 (5.50) | 1.52 (2.70) | 1.90 (2.98) |
| 30 – 39 years | N = 404 | 3.27 (5.18) | 1.37 (2.46) | 1.90 (2.93) |
| 40 – 49 years | N = 432 | 3.20 (5.07) | 1.29 (2.39) | 1.91 (2.90) |
| 50 – 59 years | N = 500 | 4.07 (5.83) | 1.74 (2.93) | 2.34 (3.11) |
| 60 – 69 years | N = 395 | 4.19 (5.97) | 1.88 (2.99) | 2.31 (3.21) |
| ≥70 years | N = 370 | 4.40 (5.67) | 2.12 (3.00) | 2.28 (2.93) |
| **Men** | **N = 1237** | **3.23 (5.02)** | **1.43 (2.49)** | **1.80 (2.73)** |
| <30 years | N = 180 | 2.90 (5.04) | 1.34 (2.45) | 1.56 (2.76) |
| 30 – 39 years | N = 222 | 2.61 (4.58) | 1.15 (2.23) | 1.45 (2.50) |
| 40 – 49 years | N = 205 | 2.61 (4.03) | 1.01 (1.84) | 1.60 (2.42) |
| 50 – 59 years | N = 246 | 3.69 (5.35) | 1.60 (2.75) | 2.09 (2.81) |
| 60 – 69 years | N = 201 | 3.83 (5.86) | 1.72 (2.88) | 2.11 (3.18) |
| ≥70 years | N = 183 | 3.75 (4.95) | 1.79 (2.55) | 1.96 (2.62) |
| **Women** | **N = 1230** | **4.29 (6.02)** | **1.86 (3.01)** | **2.42 (3.25)** |
| <30 years | N = 188 | 3.88 (5.87) | 1.68 (2.92) | 2.20 (3.14) |
| 30 – 39 years | N = 181 | 4.10 (5.76) | 1.65 (2.70) | 2.45 (3.31) |
| 40 – 49 years | N = 226 | 3.73 (5.82) | 1.54 (2.79) | 2.19 (3.26) |
| 50 – 59 years | N = 254 | 4.44 (6.25) | 1.87 (3.10) | 2.57 (3.35) |
| 60 – 69 years | N = 194 | 4.57 (6.06) | 2.05 (3.11) | 2.52 (3.23) |
| ≥70 years | N = 187 | 5.03 (6.25) | 2.44 (3.37) | 2.59 (3.18) |

Table S2. Demoralization Scale-II mean and standard deviation by age group and gender. M, mean; SD, standard deviation. DS-II, Demoralization Scale-II.
